# Supplementary material for: Income inequality and mental health in adolescents during COVID-19, results from COMPASS 2018–2021
Source: PLoS One. 2023 Oct 24;18(10):e0293195. doi: 10.1371/journal.pone.0293195 (PMC10597521; doi:10.1371/journal.pone.0293195)
Supplement: Attachment — Submitted filename: response to reviewer sep15.docx [file pone.0293195.s001.docx]

**Response to Editor**

**Editor:**

Please review your reference list to ensure that it is complete and correct. If you have cited papers that have been retracted, please include the rationale for doing so in the manuscript text, or remove these references and replace them with relevant current references. Any changes to the reference list should be mentioned in the rebuttal letter that accompanies your revised manuscript. If you need to cite a retracted article, indicate the article’s retracted status in the References list and also include a citation and full reference for the retraction notice.

**Response:** We have revisited the reference list for our manuscript and confirm that, to our knowledge, no papers cited have been retracted. Thank you for your comment.

**Response to Reviewers**

**Reviewer #2:**

[R]esearchers should carefully select a single appropriate venue for their work and adhere to the publication policies and guidelines of that journal or conference. It is crucial to prioritize transparency, integrity, and responsible conduct when disseminating research findings. Many journals require authors to transfer their copyright or grant exclusive publication rights. Dual publication violates these agreements, as it involves submitting the same work to multiple venues without proper authorization.

**Response:** Thank you for voicing your concerns re: dual publication. This manuscript has only been submitted to PLOS One, in line with journal standards. In terms of transparency, we have amended our data availability statement to align with the journal standards. Please note that the data used for our study cannot be included with our submission because of concerns with anonymity of the respondents. We have provided contact information for COMPASS data access, should this reviewer or any reader be interested in garnering access to the data used in this study. Please see page 2, paragraph 2 in the revised text.

Data availability. Data cannot be shared publicly because of ethical and confidentiality concerns. Data are available from the COMPASS team at the University of Waterloo (contact via sherry.rezvani@uwaterloo.ca) for researchers who meet the criteria for access to confidential data.
